# Housing with support for older people: a mixed-methods systematic review protocol

**DOI:** 10.12688/hrbopenres.13124.2

**Published:** 2020-11-11

**Authors:** Camille Coyle, Sarah Buggy, Olivia Cagney, Louise Farragher, Caitriona Lee, Darren Patje, Jean Long

**Affiliations:** 1 Health Research Board, Dublin, Ireland; 2 Trinity College Dublin, Dublin, Ireland

**Keywords:** systematic review, mixed-methods, protocol, housing, older people

## Abstract

**Background: **The implementation of housing with support is rapidly expanding, particularly as life expectancy is increasing throughout the world. This expansion is likely to intensify in the context of coronavirus disease 2019 (COVID-19), which has revealed the risks of relying primarily on nursing homes. This mixed-methods systematic review aims to: 1) explore older people’s perceptions and experiences of housing with support and 2) examine the impact of providing housing with support for older people on their quality of life.

**Methods: **The databases Ovid Medline, Ovid Social Policy & Practice, EBSCO CINAHL, and EBSCO SOCIndex will be searched, and grey literature will also be identified. Quality assessment will be carried out using Joanna Briggs Institute’s Critical Appraisal Checklist for Qualitative Research as well as a tool from the National Institutes of Health for observational cohort studies. This review will employ convergent parallel design; as such, qualitative and quantitative findings will be synthesised separately in the initial stage of analysis. The results from the qualitative and quantitative syntheses will then be integrated in the final stage of the analysis.

**Conclusion: **This systematic review will synthesise the evidence regarding older people’s perceptions and experiences of housing with support and the impact of providing housing with support for older people on their quality of life.

## Background

Housing plays a fundamental role in quality of life, particularly for groups of people who spend more time in the home, including older people ^1^. It is also well established that older people prefer to age and die in place ^2^. Yet, the quality and appropriateness of an older person’s home environment modulates the extent to which they can take care of themselves or be cared for at home ^3^. Housing with support aims to enable older people to age with dignity and independence by providing accessible housing and by connecting them with services that meet their social and healthcare needs ^4^. Moreover, housing with support may have the potential to reduce hospitalisation and institutionalisation among older people ^4^.

The implementation of housing with support is rapidly expanding, particularly as life expectancy is increasing throughout the world. This expansion of housing with support is likely to intensify in the context of coronavirus disease 2019 (COVID-19), which has revealed the risks of relying primarily on nursing homes. Efforts to reduce reliance on nursing homes and shift towards housing with support ought to be informed by both qualitative and quantitative evidence. In order to ensure that housing with support meets the needs of the population it aims to serve; we need to explore the perceptions and experiences of older people themselves. Doing so will allow those involved in policy and practice to incorporate the preferences of older people, which could contribute to efforts to increase demand for housing with support. Additionally, it is imperative that the expansion of housing with support be informed by a systematic and comprehensive assessment of its impact thus far. Addressing these key knowledge gaps would make an important contribution to the literature and to public policy.

Therefore, this mixed-methods systematic will examine the following research questions: 1) What are older people’s perceptions and experiences of housing with support? and 2) What is the impact of providing housing with support for older people on their quality of life? In this systematic review, ‘housing with support’ will be defined as purpose-built, non-institutional accommodation where older people have their own front door and personal living quarters and where support or care services are available. Support services could include health and social well-being programmes and/or a volunteer programme. It could also include help with housekeeping, gardening, and general maintenance. In addition, care services could include assistance with activities of daily living, basic nursing care, and help with medication.

## Methods

### Study design

This mixed-methods review will employ convergent parallel design. We chose this approach rather than a sequential approach because convergent parallel design is considered more appropriate for reviews addressing separate qualitative and quantitative research questions ^5^. As such, in accordance with convergent parallel design qualitative and quantitative data will be analysed separately, and the findings will then be integrated ^5^. By considering qualitative and quantitative studies in isolation prior to synthesising them, we will be able to do justice to each of the two overarching research paradigms before integrating our results.

### Search strategy

The search strategy that was designed for this review is based around three overarching concepts – older people, models of housing with support, and mixed-methods research. One comprehensive search of the published, peer-reviewed research on housing with support for older people will be used to answer both research questions. Preliminary scoping searches indicated that research articles informing both research questions were to be found across a range of sources, including medical and sociological sources. The search strategy was initially developed for the MEDLINE (Ovid) database and was subsequently translated for use in the CINAHL, SocINDEX (both on the EBSCO platform), and Social Policy & Practice (Ovid) databases (see extended data for the full search strategy ^6^). These databases cover a range of subject areas, professions, and geographical areas, ultimately providing a wide scope of research sources.

No language limits were applied to the search strategy. Following trial searches of MEDLINE and the examination of references from previously published reviews, the decision was taken to limit the search to articles published from the year 2000 to present. For the purposes of this review, older people have been defined as adults aged 50 years and over, so where possible, limits to include only research on older people were applied.

A separate grey literature search will also be undertaken to inform each of the two research questions, based on key terms used for the database search and using the same inclusion and exclusion criteria that will be used for the database search.

The search strategy was developed by an information specialist (LF) in consultation with the research team. It was reviewed and modified following consultation with another information specialist on the team (CL).

### Supplementary search strategies

The following supplementary search strategies will be used:

- Reference checking: The reference section of each included article will be checked for relevant articles.
- Citation chasing: Articles that cited the included articles will be checked for relevance.

These strategies will be used for each included study, including studies found through the supplementary search strategies and through the grey literature search. In addition, any systematic reviews that are identified at any stage in the screening process will be targeted and their reference sections will be checked for relevant studies.

### Screening

All database search results will be imported into EPPI-Reviewer 4 for title and abstract screening for each research question. Therefore, the search results will be screened in full twice. This will ensure that screeners are able to focus on identifying relevant studies for each research question in isolation. EPPI-Reviewer’s priority screening function will be used to improve the efficiency of title and abstract screening ^7^.

### Study selection

For the first research question, focusing on older people’s perceptions and experiences of housing with support, studies will be selected according to the criteria outlined in Table 1. Only studies that used one of five widely recognised qualitative methodologies – case study research, ethnography, grounded theory research, narrative analysis, and phenomenology – as outlined by Creswell & Poth (2017) ^8^ will be considered for inclusion. Qualitative methodologies are often ill-defined, and we feel that it is important to limit our included studies to those that specify a methodology and, moreover, use a widely recognised methodology.

**Table 1.** Eligibility criteria for the perceptions and experiences research question.

Domain	Inclusion criteria	Exclusion criteria
Population	People aged 50 and over	Family members and carers
Intervention	Purpose-built housing for older people, where they have their own front door with personal living quarters and where support services are available on site.	Adaptations to the family home Age-friendly cities Age-friendly neighbourhoods Hospitals Housing purpose-built for homeless older people, blind older people, older people with dementia or disabilities Housing with shared rooms Inpatient care centres Naturally occurring retirement communities Nursing homes Skilled nursing facilities
Study design *	Grounded theory research Ethnographic research Phenomenological research Qualitative case studies Narrative analyses	Conceptual or theoretical articles Conference abstracts Letters to the editor Opinion pieces Books
Publication date	2000-present	

*Only studies that used one of five widely recognised qualitative methodologies outlined by Creswell & Poth (2017) ^8^ will be considered for inclusion.

For the second research question, focusing on the impact of housing with support for older people, studies will be selected according to the criteria outlined in Table 2. Quantitative studies will not be excluded based on outcome or comparison group, because we did not want to risk excluding studies with outcomes that we may not have considered.

**Table 2.** Eligibility criteria for the impact research question.

Domain	Inclusion criteria	Exclusion criteria
Population	People aged 50 and over	Family members and carers
Intervention	Purpose-built housing for older people, where they have their own front door, with personal living quarters and where support services are available on site.	Adaptations to the family home Age-friendly cities Age-friendly neighbourhoods Hospitals Housing purpose-built for homeless older people, blind older people, older people with dementia or disabilities Housing with shared rooms Inpatient care centres Naturally occurring retirement communities Nursing homes Skilled nursing facilities
Study design	Quantitative studies with at least two data collection time points	Conceptual or theoretical articles Conference abstracts Letters to the editor Opinion pieces Books
Publication date	2000-present	

Disagreements regarding inclusion for both research questions will be resolved through discussion until consensus is reached.

### Quality assessment and confidence in evidence

For the first research question, focusing on older people’s perceptions and experiences of housing with support, Joanna Briggs Institute’s Critical Appraisal Checklist for Qualitative Research will be used ^9^. Additionally, the CERQual (Confidence in the Evidence from Reviews of Qualitative Research) approach will be used to assess confidence in qualitative findings. The CERQual method enables reviewers to transparently assess and describe the extent to which a review finding is a reasonable representation of the phenomenon of interest, such that the phenomenon of interest is unlikely to be substantially different from the research finding ^10^. It includes four elements: 1) methodological limitations; 2) relevance to the review question; 3) coherence; and 4) adequacy of data ^10^. These elements are used to assess overall confidence in qualitative findings. There are four levels of confidence: high, moderate, low, or very low ^10^.

For the second research question, focusing on the impact of housing with support, quality assessment will be carried out using a tool for observational cohort studies from the National Heart, Lung, and Blood Institute of the National Institutes of Health in the United States of America ^11^. This tool uses 12 items to assess quality. We will also use *British Medical Journal* guidelines to assign levels of evidence to each quantitative study ^12^, and the GRADE certainty of evidence tool ^13^ to write our strength of evidence recommendation for quantitative findings. In the GRADE tool, the levels of evidence range from one to four. The certainty of evidence can be high, moderate, low, or very low. The quality of evidence drives the strength of recommendation, which is one of the last translational steps of research and is most proximal to patient care.

For both research questions, each included study will be independently quality assessed by two reviewers, with any disagreements being resolved by consensus. Quality assessment results will not be used to exclude studies.

### Data extraction

Data will be extracted by a single reviewer into a bespoke extraction sheet for each research question (see extended data ^6^). For the research question focusing on older people’s perceptions and experiences of housing with support, the following data will be extracted: first author and year of publication, study title, country, housing model, study design, data collection method, dates of data collection, population age, number of participants, proportion of males and females, and findings. For the quantitative research question focusing on the impact of housing with support, the following data will be extracted: first author and year of publication, study title, country, housing model, study design, timepoints, participants at baseline, participants at follow-up, loss to follow-up, population age, proportion of males and females, and findings.

Extracted data will be verified independently by a second reviewer. Journal websites for the included articles will be checked for supplementary data and errata, and authors will be contacted if data required for quality appraisal and analysis are missing.

### Data analysis

Thematic synthesis will be used to integrate the results of the included qualitative studies. Thematic synthesis has three stages: 1) line-by-line coding of text; 2) the development of descriptive themes; 3) and the generation of analytical themes ^14^. We will use Dedoose (2020) software to code text and to develop our descriptive themes, which we will draw inductively from the included studies. The generation of analytical themes represents the stage of synthesis whereby reviewers integrate the primary studies and generate novel interpretations of findings ^14^. We will develop analytical themes by revisiting the descriptive themes in light of the qualitative research question – what are older people’s perceptions and experiences of housing with support?

For the quantitative research question focusing on the impact of housing with support, we will conduct a feasibility assessment to identify the level of heterogeneity among the included quantitative studies, which will determine whether or not meta-analysis is suitable. A core assumption underpinning meta-analysis is that the studies being pooled are homogeneous; therefore, all sources of heterogeneity and variation must be assessed before conducting a meta-analysis. Our feasibility analysis will consider population, comparator, intervention, measurement scale, and length of time to follow-up. If meta-analysis is feasible, R software (2020) will be used, and we will conduct a meta-analyses for main outcomes as well as sensitivity and subgroup analysis by age, gender, and socio-economic groups. If meta-analysis is not feasible, we will present a narrative synthesis of the included quantitative studies using their summary statistics and vote counting.

### Dissemination of information

Findings will be disseminated as peer-reviewed publications.

### Study status

The database search was conducted in November 2019. Full-text screening was completed in May 2020. It is anticipated that the review will be completed in November 2020.

## Conclusion

This systematic review will synthesise the evidence regarding older people’s perceptions and experiences of housing with support and the impact of providing housing with support for older people.

## Declarations

### Data availability

***Underlying data.*** No data are associated with this article

***Extended data.*** Open Science Framework: Housing with support for older people: a mixed-methods systematic review protocol. https://doi.org/10.17605/OSF.IO/YU7S6 ^6^

This project contains the following extended data:

- Search strategy.docx (study search strategy)
- Data extraction sheets.docx (Sheet used for data extraction)

***Reporting guidelines.*** Open Science Framework: PRISMA-P checklist for ‘Housing with support for older people: a mixed-methods systematic review protocol’ https://doi.org/10.17605/OSF.IO/YU7S6 ^6^

Data are available under the terms of the Creative Commons Zero "No rights reserved" data waiver (CC0 1.0 Public domain dedication).

## References

[1] Centre for Ageing Research and Development in Ireland: Focus on housing and the health of older people. Belfast: Centre for Ageing Research and Development in Ireland.2013 Reference Source

[2] Friedland RB Summer L: Demography is not destiny, revisited. New York, NY: The Commonwealth Fund;2005 Reference Source

[3] Cullen K Delaney S Dolphin C: The role and future development of supportive housing for older people in Ireland. Dublin, Ireland: National Council on Ageing and Older People;2007 Reference Source

[4] Ewen HH Lewis DC Carswell AT: A model for aging in place in apartment communities. *J Hous Elderly.* 2017;31(1):1–13. 10.1080/02763893.2016.1268555

[5] Hong QN Pluye P Bujold M: Convergent and sequential synthesis designs: implications for conducting and reporting systematic reviews of qualitative and quantitative evidence. *Syst Rev.* 2017;6(1):61. 10.1186/s13643-017-0454-2 28335799 PMC5364694

[6] Coyle C: Housing with support for older people: a mixed-methods systematic review protocol.2020 10.17605/OSF.IO/YU7S6 PMC7592024 33145477

[7] Eppi Centre: EPPI-Reviewer 4: software for research synthesis. User manual. London: EPPI-Centre;2018 Reference Source

[8] Creswell JW Poth CN: Qualitative Inquiry and Research Design: Choosing Among Five Approaches. New York: SAGE Publications;2017 Reference Source

[9] The Joanna Briggs Institute: The Joanna Briggs Institute critical appraisal tools for use in JBI systematic reviews. Checklist for qualitative research. Adelaide: The Joanna Briggs Institute;2017 Reference Source

[10] Lewin S Bohren M Rashidian A: Applying GRADE-CERQual to qualitative evidence synthesis findings—paper 2: how to make an overall CERQual assessment of confidence and create a Summary of Qualitative Findings table. *Implement Sci.* 2018;13(Suppl 1):10. 10.1186/s13012-017-0689-2 29384082 PMC5791047

[11] NIH National Heart LaBI: Study quality assessment tools. Quality assessment tool for observational cohort and cross-sectional studies. Bethesda, Maryland: NIH National Heart, Lung and Blood Institute; [No date]. Reference Source

[12] Murad MH Asi N Alsawas M: New evidence pyramid. * Evid Based Med.* 2016;21(4):125–7. 10.1136/ebmed-2016-110401 27339128 PMC4975798

[13] Schünemann H Brożek J Guyatt G: Overall confidence in estimates of effects. In: *GRADE handbook for grading quality of evidence and strength of recommendations* Grading of Recommendations Assessment, Development and Evaluation (GRADE) Working Group,2013 Reference Source

[14] Thomas J Harden A: Methods for the thematic synthesis of qualitative research in systematic reviews. * BMC Med Res Methodol.* 2008;8(1):45. 10.1186/1471-2288-8-45 18616818 PMC2478656

