# Supplementary material for: Housing with support for older people: a mixed-methods systematic review protocol
Source: HRB Open Res. 2020 Nov 11;3:64. Originally published 2020 Sep 11. [Version 2] doi: 10.12688/hrbopenres.13124.2 (PMC7592024; doi:10.12688/hrbopenres.13124.2)
Supplement: Peer review history

# Peer review: Reviewer response for version 2

**DOI:** 10.21956/hrbopenres.14306.r28325

**Published:** 2020-11-11

**Authors:** Lynne S. Nemeth

**Affiliations:** 1 College of Nursing, Medical University of South Carolina, Charleston, SC, USA

No further comments. Thank you.

Is the study design appropriate for the research question?

Yes

Is the rationale for, and objectives of, the study clearly described?

Yes

Are sufficient details of the methods provided to allow replication by others?

Yes

Are the datasets clearly presented in a useable and accessible format?

Not applicable

Reviewer Expertise:

My area of research is related to primary care, health information technologies, mhealth applications and implementation science.

I confirm that I have read this submission and believe that I have an appropriate level of expertise to confirm that it is of an acceptable scientific standard.

# Peer review: Reviewer response for version 1

**DOI:** 10.21956/hrbopenres.14234.r28022

**Published:** 2020-10-27

**Authors:** France Légaré, Lionel Adisso

**Affiliations:** 1 Canada Research Chair in Shared Decision Making and Knowledge Translation, Laval University, Quebec City, Canada; 2 Canada Research Chair in Shared Decision Making and Knowledge Translation, Laval University, Quebec City, SC, USA

This paper reports a study protocol for a mixed-methods systematic review that explores perceptions and experiences of older adults about housing with support and examines the impact of providing housing with support on older adult’s quality of life. The study protocol is reported according to PRISMA-P. The rationale is well developed and the objectives are clearly articulated. However, it will be useful to provide references in paragraph 2 (background) to support the interesting ideas elaborated (for example, about quantitative and qualitative evidence). It will also be useful to provide more clarification about the definition of “housing with support” and a reference if available. It will be useful for better understanding of the rationale.

 The systematic review method is globally appropriate for the research questions. The eligibility criteria are clearly defined, information sources are well described, search strategy are well elaborated, data management and the study selection process are well described. However, we suggest more details and justification about the study design to help readers, interested, to well understand your choices (for example why authors choose a convergent design and not a sequential one?). As recommended, a pair-screened study selection process and an explicit data extraction are planned. In addition, anticipated methods for assessing quality of assessment and confidence in evidence are clearly described (e.g. GRADE, CERQual). Data analysis described how qualitative and quantitative studies will be analyzed. A thematic approach will be used for qualitative studies synthesis and *Dedoose, 2020* software will be used to perform the analysis. After assessing heterogeneity of quantitative studies, depending on the results of this assessment, a meta-analysis (using *R* Software) or a narrative synthesis is planned. The authors planned to evaluate the impact of the covariates. Unfortunately, they do not specify how this impact would be evaluated (by sensitivity analyses, subgroup analyses or other?). It might be useful to provide a final precision in order to refine the wonderful work that has been started. In total, authors provided enough details about the methods to allow replication.

 Since the study is a protocol, the data extraction sheet which is an overview of the datasets is well presented, useable and in accessible format (OSF Registration).

Is the study design appropriate for the research question?

Yes

Is the rationale for, and objectives of, the study clearly described?

Yes

Are sufficient details of the methods provided to allow replication by others?

Yes

Are the datasets clearly presented in a useable and accessible format?

Yes

Reviewer Expertise:

My area of research is related to shared decision making, knowledge translation, housing options for older people, sex and gender, epidemiology, biostatistics.

We confirm that we have read this submission and believe that we have an appropriate level of expertise to confirm that it is of an acceptable scientific standard.

# Response

**Published:** 2020-10-28

**Authors:** Camille Coyle

**Affiliations:** Health Research Board, Ireland

**Thank you very much for your review of our protocol. Regarding your suggestion that we add references to the second paragraph of the background section, we have not done so because these sentences reflect original ideas regarding our justification for conducting the review. We agree that the protocol would benefit from further clarification regarding our definition of housing with support, so we have provided more detail. We also agree that further detail regarding our selection of convergent parallel design is important, therefore we have added to this section. Finally, we have added detail to our quantitative data analysis section, as suggested. Thank you again for your review of our protocol, we greatly appreciate your time and expertise.**

# Peer review: Reviewer response for version 1

**DOI:** 10.21956/hrbopenres.14234.r28021

**Published:** 2020-10-14

**Authors:** Lynne S. Nemeth

**Affiliations:** 1 College of Nursing, Medical University of South Carolina, Charleston, SC, USA

This paper reports a study protocol for a systematic review that evaluates perceptions/experiences of older adults related to housing with support, and quality of life impacts of housing with support for older adults. The rationale and objectives for this review were clearly stated. However, in the definition of housing with support, it is possible that one group of older adults may be excluded that would be relevant - that is the setting of assisted living facilities that provide meals and activities for seniors in a setting where the residents have a mini-kitchen but not cooking facilities in their apartments. This is a common model for older adults to receive support in their housing arrangements in the US.

 The systematic review approach is appropriate for the research questions. The search strategy is transparent in text, with additional data provided in Open Science Framework documents. Inclusion criteria were clearly stated. Joanna Briggs Institute Critical Appraisal Criteria, and CERQual for qualitative research criteria will be used to assess quality of the evidence for the perceptions and experiences research question. For the quality of life question, NHLBI criteria for observational cohort studies will be used, along with BMJ guidelines and GRADE tool to assign levels of evidence. Independent assessment by two raters is planned. Data extraction will be conducted by a single reviewer with a clear data extraction sheet that is verified by a second reviewer. A thematic analysis will be used to integrate results of the studies, using Dedoose software. A feasibility assessment will be used to identify whether a meta-analysis is appropriate.  These details of methods are sufficient to permit replication of this study.

 As this is a study protocol, and the data are not yet provided, the dataset usability and accessibility criteria do not seem relevant to this review.

Is the study design appropriate for the research question?

Yes

Is the rationale for, and objectives of, the study clearly described?

Yes

Are sufficient details of the methods provided to allow replication by others?

Yes

Are the datasets clearly presented in a useable and accessible format?

Not applicable

Reviewer Expertise:

My area of research is related to primary care, health information technologies, mhealth applications and implementation science.

I confirm that I have read this submission and believe that I have an appropriate level of expertise to confirm that it is of an acceptable scientific standard.

# Response

**Published:** 2020-10-28

**Authors:** Camille Coyle

**Affiliations:** Health Research Board, Ireland

**Thank you very much for your review of our protocol, we greatly appreciate your time and expertise. Regarding your suggestion that the criteria of cooking facilities could exclude older people in assisted living communities in the US who have their own mini kitchens, thank you for bringing this to our attention. We have removed cooking facilities from the inclusion criteria to ensure that this population will be included if relevant articles are identified.**
